# Hepatitis B screening among asylum seekers: a retrospective cohort study in Modena, Northern Italy

**DOI:** 10.1093/eurpub/ckag188

**Published:** 2026-10-21

**Authors:** Riccardo Mazzoli, Lucia Borsari, Maria La Torre, Zaynalabedin Kahfian, Alessandra Fantuzzi, Marco Vinceti, Annalisa Bargellini, Tommaso Filippini, Stefania Paduano

**Affiliations:** Department of Biomedical, Metabolic and Neural Sciences, University of Modena and Reggio Emilia, Modena, Italy; Environmental, Genetic and Nutritional Epidemiology Research Center (CREAGEN), Department of Biomedical, Metabolic and Neural Sciences, University of Modena and Reggio Emilia, Modena, Italy; Public Hygiene Service, Department of Public Health, AUSL Modena, Modena, Italy; Department of Biomedical, Metabolic and Neural Sciences, University of Modena and Reggio Emilia, Modena, Italy; Public Hygiene Service, Department of Public Health, AUSL Modena, Modena, Italy; Public Hygiene Service, Department of Public Health, AUSL Modena, Modena, Italy; Department of Biomedical, Metabolic and Neural Sciences, University of Modena and Reggio Emilia, Modena, Italy; Environmental, Genetic and Nutritional Epidemiology Research Center (CREAGEN), Department of Biomedical, Metabolic and Neural Sciences, University of Modena and Reggio Emilia, Modena, Italy; Department of Epidemiology, Boston University School of Public Health, Boston, MA, United States; Department of Biomedical, Metabolic and Neural Sciences, University of Modena and Reggio Emilia, Modena, Italy; Department of Biomedical, Metabolic and Neural Sciences, University of Modena and Reggio Emilia, Modena, Italy; Environmental, Genetic and Nutritional Epidemiology Research Center (CREAGEN), Department of Biomedical, Metabolic and Neural Sciences, University of Modena and Reggio Emilia, Modena, Italy; School of Public Health, University of California Berkeley, Berkeley, CA, United States; Department of Biomedical, Metabolic and Neural Sciences, University of Modena and Reggio Emilia, Modena, Italy

## Abstract

Hepatitis B virus remains a major health challenge among migrants from high-endemic regions arriving in low-endemic countries like Italy. However, more evidence on both infection burden and immunity gaps among asylum seekers is needed to inform prevention policies. We conducted a retrospective cohort study of 739 asylum seekers screened for Hepatitis B virus (HBV) in Modena, Northern Italy, between 2019 and 2023. Serological testing included quantitative determination of HBsAg, HBcAb, and HBsAb, allowing identification of active infection, natural immunity, vaccine-induced immunity, and susceptibility. Demographic characteristics, geographic origin, and migration routes were analyzed in relation to HBV status. Participants were predominantly male (94.7%), with a mean age of 27.2 years, and originated mainly from South Asia (59.4%) and Sub-Saharan Africa (26.1%). Overall HBsAg prevalence was 4.1%, substantially higher than what reported for the Italian general population (<1%). Prevalence was highest among Sub-Saharan African migrants (7.3%), who also showed the greatest proportion of natural immunity (36.3%), while South Asian migrants had a prevalence of 3.6% and Northern African migrants null. Among HBsAg-negative individuals, 57% were susceptible to HBV infection, particularly those from South Asia and Northern Africa. Susceptibility was associated with male sex (OR 4.08, 95% CI 1.34–12.44) and age ≥25 years (OR 2.38, 95% CI 1.40–4.04), consistent with birth before the introduction of universal HBV vaccination in many countries. Most HBsAg-positive individuals reported transit through Libya. Asylum seekers in Italy show a high prevalence of HBV infection alongside substantial immunity deficits. Systematic screening and targeted catch-up vaccination should be prioritized within migrant health services as a key public health intervention.

## Introduction

Hepatitis B virus (HBV) remains a major global health challenge, with an estimated over 300 million people chronically infected worldwide and a HBsAg prevalence of approximately 3.6%–4.1% as of 2019 [1, 2]. The burden is unevenly distributed geographically, with higher prevalence in Sub-Saharan Africa, East Asia, and parts of the Middle East, regions that also account for the majority of HBV-related deaths [1, 2]. In contrast, Italy is a low-endemicity country, with HBsAg prevalence in the general population estimated at or below 1% in recent years [3, 4].

However, migration from high-endemic regions such as sub-Saharan Africa and East Asia is a major driver of the persistence and potential re-emergence of HBV in low-endemic countries, such as Italy [5, 6]. A substantial proportion of new HBV diagnoses appears to occur in non-native populations, with migrants accounting for a major share of chronic HBV cases, especially relative to their population size [3, 6–8]. Furthermore, migrants are a key at-risk group, given that their HBV status is often unknown or unmanaged. Barriers such as limited access to healthcare, stigma, and low health literacy frequently result in delayed diagnosis and suboptimal linkage to care. Many migrants remain undiagnosed for years after arrival, increasing the risk of advanced liver disease and viral transmission [5, 8].

Early identification of HBV carriers through targeted screening enables timely linkage to care, monitoring of liver function, assessment for antiviral therapy, preventing disease progression, and reducing transmission risk within households and communities. As undiagnosed chronic HBV infection is frequently asymptomatic for years, without targeted screening migrant HBV-carrier individuals risk remaining invisible to health systems [9, 10].

Hepatitis B vaccination is a cornerstone of HBV prevention in Italy, where universal childhood immunization has been in place since 1991, resulting in high coverage and low endemicity among the native population. The Italian National Vaccination Plan (Piano Nazionale Prevenzione Vaccinale, PNPV) explicitly includes targeted vaccination measures for migrants, irrespective of age, to address health inequalities and elevated infectious disease risk, including free HBV vaccination for household contacts of HBsAg-positive individuals. National guidelines for the health screening of newly arrived migrant recommend HBV serological testing as part of the initial health assessment [11]. Nevertheless, the implementation of these recommendations varies substantially across Italian regions and within regions, particularly regarding the systematic offer of free HBV vaccination to susceptible asylum seekers and their contacts in reception centers. However, many migrants, especially adults, arrive from countries with widely variable and often suboptimal HBV vaccine coverage, leaving a substantial proportion susceptible to infection [12–14]. Catch-up vaccination and serological testing in migrant reception centers represent a critical opportunity for HBV prevention among migrants and allow for targeted vaccination, aligning with WHO’s hepatitis elimination goals (2030) [15]. In response to these needs, the Italian NHS and the Public Health Department of the Local Health Authority (AUSL) of Modena have implemented a structured screening program for asylum seekers, including HBV serological testing and targeted vaccination.

This study aims to evaluate HBV infection status and susceptibility in a large cohort of asylum seekers hosted in the province of Modena between 2019 and 2023. We further examine socio-demographic characteristics and country of origin to identify groups at higher vulnerability, thereby informing public health strategies aimed at improving immunization coverage and long-term disease prevention in migrant populations.

## Methods

### Study population

This retrospective cohort study included a subset of asylum seekers assessed by the AUSL of Modena from March 2019 to November 2023 who underwent screening for sexually transmitted infection, including HBV. All individuals included in the study were newly arrived asylum seekers referred for an initial health screening. Participation in the screening program was part of the voluntary health assessment by the AUSL of Modena according to regional (Emilia-Romagna Region D.G.R. n. 1304/2022) and national guidelines [11]; refusal did not affect access to any health or social services. Nonetheless, participation rates were high, supported by the active involvement of Non-Governmental Organizations (NGOs) in coordinating appointments and explaining the opportunity to receive free health assessment and treatment.

The study was approved by the Area Vasta Emilia Nord Ethics Committee (approval no. AOU0033904/23 on 15 November 2023). All participants signed an individual informed consent for health assessment, with the help of a cultural mediator. Individual informed consent for the study participation was waived by the Ethics Committee, given the retrospective nature of the study, the use of fully anonymized data routinely collected within the existing public health surveillance framework, and the absence of any additional intervention beyond standard clinical practice.

### Clinical assessment and data collection

Health assessments took place at public health outpatient clinics across the province of Modena. Each clinical visit was conducted by a physician and a health assistant, with a cultural mediator always present to facilitate communication. During the initial visit, a standardized interview was conducted to collect demographic data (e.g. sex, date of birth, country of origin), migration history (e.g. departure date, countries crossed, date, and place of arrival in Italy), and past and current medical history. Countries of origin were further grouped according to the area of origin (Table S1). A full physical examination was carried out, which included the collection of anthropometric and vital parameters and a battery of tests for communicable diseases. A blood sample was also collected to test for HBV, Hepatitis C (HCV), syphilis, and human immunodeficiency virus (HIV).

For HBV specifically, serological markers included hepatitis B surface antigen (HBsAg) to identify active infection, hepatitis B core antibody (HBcAb) to assess past exposure, and hepatitis B surface antibody (HBsAb) to evaluate immune protection. Individuals with HBsAb levels below 3 mIU/ml were considered susceptible and were offered a full vaccination course consisting of three doses. Those with HBsAb levels between 3 and 10 mIU/ml were considered operationally classified as partially protected, on the assumption that a sub-threshold anti-HBs level may indicate residual immune memory rather than complete susceptibility [16], and received a booster dose. This classification, in the absence of documented vaccination history, should be interpreted as an operational guide to clinical decision-making rather than a confirmed immunological determination. Subjects with HBsAb levels above 10 mIU/ml were deemed adequately immunized and required no further intervention [16]. Those with active infections or needing secondary-level care were referred through dedicated care pathways within the local health system. HBeAg was examined to assess viral replication status and infectiousness and past resolved infections were defined by isolated anti-HBc (HBcAb) positivity (HBsAg negative). Specifically, vaccine-induced immunity was defined as HBsAb >10 mIU/ml with HBcAb negative, reflecting antibody levels consistent with prior vaccination in the absence of any marker of natural infection; natural immunity was defined as HBcAb positivity in HBsAg-negative individuals, indicating prior natural HBV infection regardless of HBsAb level. Individuals with isolated HBcAb positivity were subject to the same HBsAb-based vaccination algorithm as all other participants; HBcAb status was used exclusively to classify the nature of existing immunity (natural vs vaccine-induced) for descriptive and analytical purposes and did not modify the vaccination offer.

### Data classification

All clinical and laboratory data were systematically recorded using the “Migrant Checklist,” a standardized electronic form developed by the Emilia-Romagna Region specifically for the health assessment and surveillance of newly arrived migrants (Emilia-Romagna Region. D.G.R. n. 1304/2022). This tool ensures consistency in data collection across regional facilities and supports the integration of health surveillance with epidemiological monitoring systems.

Migration routes were reconstructed from self-reported information on countries of origin, countries crossed, and disembarkation in Italy; where discrepancies emerged, the most plausible route was inferred based on consistency with the other migratory patterns. Routes were classified into three mutually exclusive categories based on the primary pathway used to reach Italy, namely “Mediterranean Libya route,” “Mediterranean non-Libya route” (those who crossed the Mediterranean Sea to reach Italy without transiting through Libya), and “Balkan and Other overland routes.” Route classification was performed independently of serological outcomes to minimize classification bias.

### HBV vaccination policies assessment

To contextualize the observed HBV susceptibility among the study population, we conducted a grey literature review to identify national HBV vaccination policies in the countries of origin of the asylum seekers included in our sample. The search was carried out using Google, Google Scholar, and the official websites and reports of national Ministries of Health, as well as international organizations such as the World Health Organization (WHO), UNICEF, and relevant NGOs.

The review focused on the existence of universal HBV vaccination programs, the year of national implementation, the target age groups, and whether vaccination was provided free of charge. Special attention was given to assessing whether these policies were in effect during the probable childhood or adolescence of the individuals in the cohort, based on year of birth. The aim was to estimate potential prior vaccine exposure in the countries of origin and to support the interpretation of observed serological patterns.

### Data analysis

We anonymized and entered all the collected data into a secure database for analysis. Descriptive statistics were used to summarize demographic characteristics, HBV serostatus, and vaccination needs. We expressed categorical variables as frequencies and percentages, and computed means and standard deviations or medians and interquartile ranges for continuous variables. Potential collinearity between variables was formally assessed via variance inflation factor (VIF) analysis across all regression models.

All analyses were conducted using Stata version 18.0 (StataCorp LLC, College Station, TX, USA, 2023). Geospatial visualization of routes was conducted with QGIS version 3.34.15 LTR (QGIS Association, Switzerland).

## Results

Baseline demographic and clinical characteristics of the migrant population were summarized in Table 1. The study population consisted of 739 individuals, predominantly male (94.7%, *n* = 700) with a mean age of 27.2 years (SD = 7.4). The majority originated from South Asia (59.4%, *n* = 439), followed by Sub-Saharan Africa (26.1%, *n* = 193) and Northern Africa (14.2%, *n* = 105). Of those classified as South Asia, 90.4% originated from Bangladesh (*n* = 303, 69.0%) or Pakistan (*n* = 107, 24.4%), with the remainder from Afghanistan, India, and other countries. Most participants arrived in Sicily (72.8%, *n* = 538), with the Mediterranean route through Libya being the predominant entry pathway (69.0%, *n* = 510). Across the 26 countries represented, Bangladesh (41.0%, *n* = 303) and Pakistan (14.5%, *n* = 107) contributed the largest portions of the sample (Table S2). Figure 1 shows the overview of the migration routes.

**Figure 1:**
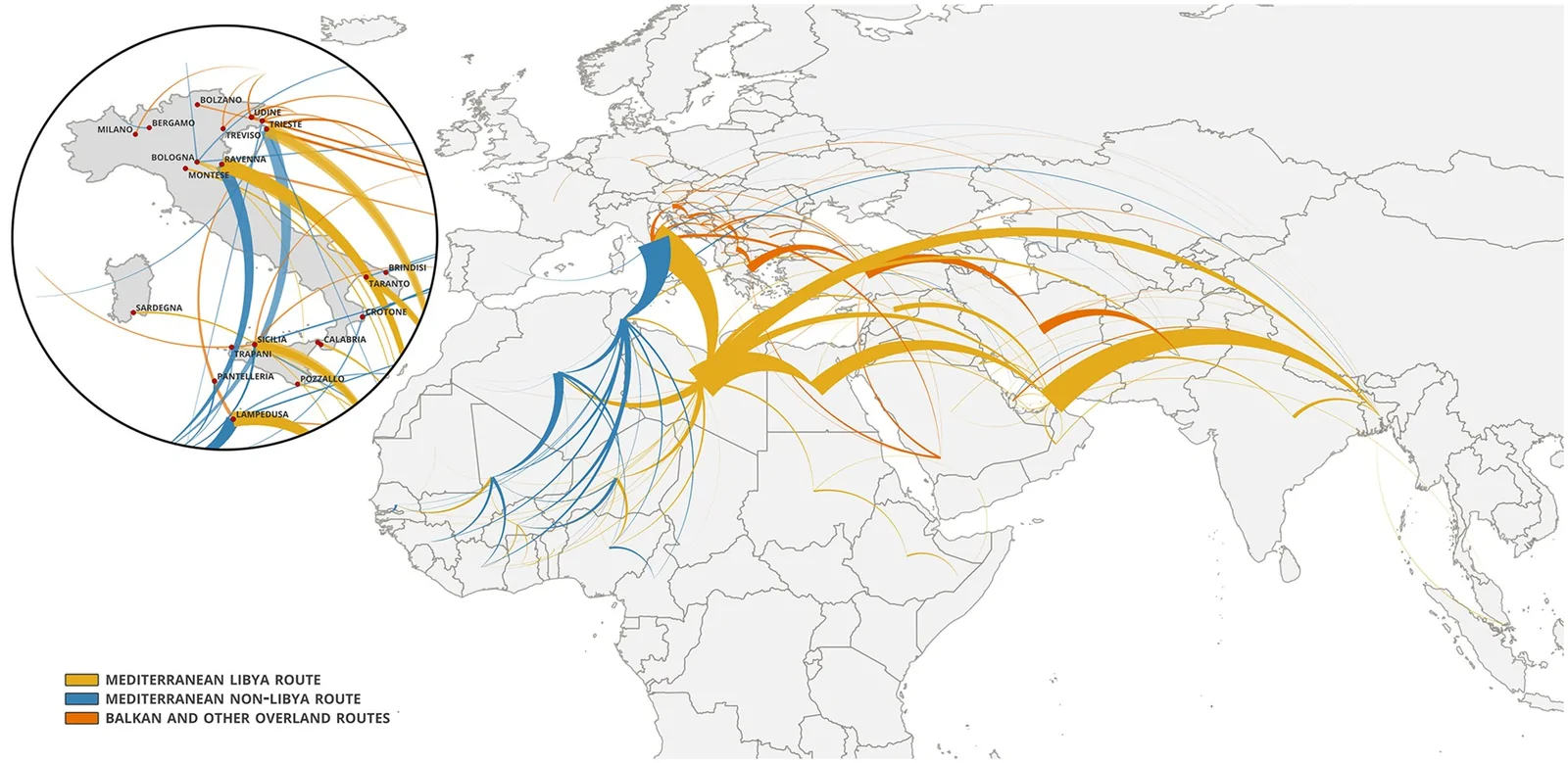
Migratory routes toward Italy. Arrows indicate main migration pathways, line thickness is proportional to the number of migrants. Mediterranean Libya route in yellow, Mediterranean non-Libya route in blue, and Balkan and other overland routes in orange. Italian entry points are shown in detail in the inset map.

**Table 1:** Demographical characteristics and travel information of the migrant population.

	*N*	%
Sex		
Male	700	94.7
Female	39	5.3
Age mean (SD) years	27.2	(7.4)
Age group		
18–20	133	18.0
21–24	216	29.2
25–29	163	22.1
30–35	104	14.1
>35	123	16.6
Macro area of origin		
South Asia	439	59.4
Sub-Saharan Africa	193	26.1
Northern Africa	105	14.2
Unknown	2	0.3
Region of arrival		
Sicily	538	72.8
Friuli-Venezia Giulia	99	13.4
Puglia	33	4.5
Emilia-Romagna	14	1.9
Calabria	14	1.9
Unknown	20	2.7
Other	21	2.9
Entry routes		
Mediterranean Libya route	510	69.0
Mediterranean non-Libya route	122	16.5
Balkan and Other overland routes	107	14.5

Table 2 presents HBsAg status, showing that 4.1% of participants (*n* = 30) tested positive, most of them being males. Sub-Saharan African migrants exhibited the highest HBsAg positivity rate (7.3%), followed by South Asian migrants (3.6%), while no participant from Northern Africa tested positive. HBsAg prevalence increased slightly with age, from 3.7% (*n* = 13) in those under 25 years to 4.4% (*n* = 17) in those aged 25 and older. Regarding entry routes, HbsAg prevalence results higher among individuals traveling through the Mediterranean Libya route (5.5% vs 1.6%).

**Table 2:** HBV serological status and immunization of the study population.

	HBsAg positive	Susceptible (HBsAb ≤2 mIU/ml)		Partially protected (HBsAb = 3–10 mIU/ml)		Adequately immunized (HBsAb >10 mIU/ml)		Total
		Negative HBcAb	Positive HBcAb	Negative HBcAb	Positive HBcAb	Negative HBcAb	Positive HBcAb	
	30 (4.1%)	400 (54.1%)	21 (2.8%)	49 (6.6%)	29 (3.9%)	77 (10.4%)	133 (18.0%)	739 (100.0%)
Sex								
Female	1 (2.6%)	13 (33.3%)	1 (2.6%)	0 (0.0%)	4 (10.3%)	6 (15.4%)	14 (35.9%)	39 (100.0%)
Male	29 (4.1%)	387 (55.3%)	20 (2.9%)	49 (7.0%)	25 (3.6%)	71 (10.1%)	119 (17.0%)	700 (100.0%)
Age								
<25 years	13 (3.7%)	186 (53.3%)	12 (3.4%)	28 (8.0%)	12 (3.4%)	49 (14.0%)	49 (14.0%)	349 (100.0%)
≥25 years	17 (4.4%)	214 (54.9%)	9 (2.3%)	21 (5.4%)	17 (4.4%)	28 (7.2%)	84 (21.5%)	390 (100.0%)
Macro area of origin								
South Asia	16 (3.6%)	283 (64.5%)	7 (1.6%)	15 (3.4%)	11 (2.5%)	46 (10.5%)	61 (13.9%)	439 (100.0%)
Sub-Saharan Africa	14 (7.3%)	49 (25.4%)	14 (7.3%)	19 (9.8%)	18 (9.3%)	9 (4.7%)	70 (36.3%)	193 (100.0%)
Northern Africa	0 (0.0%)	67 (63.8%)	0 (0.0%)	15 (14.3%)	0 (0.0%)	22 (21.0%)	1 (1.0%)	105 (100.0%)
Unknown	0 (0.0%)	1 (50.0%)	0 (0.0%)	0 (0.0%)	0 (0.0%)	0 (0.0%)	1 (50.0%)	2 (100.0%)
Entry routes								
Mediterranean Libya route	28 (5.5%)	274 (53.7%)	14 (2.7%)	37 (7.3%)	17 (3.3%)	46 (9.0%)	94 (18.4%)	510 (100.0%)
Mediterranean non-Libya route	2 (1.6%)	54 (44.3%)	6 (4.9%)	8 (6.6%)	10 (8.2%)	16 (13.1%)	26 (21.3%)	122 (100.0%)
Balkan and Other overland routes	0 (0.0%)	72 (67.3%)	1 (0.9%)	4 (3.7%)	2 (1.9%)	15 (14.0%)	13 (12.1%)	107 (100.0%)

Among HBsAg-negative individuals (*n* = 709), 54.1% (*n* = 400) were susceptible to HBV infection (HBsAb ≤2 mIU/ml and HBcAb negative), 6.6% (*n* = 49) showed partial protection (HBsAb 3–10 mIU/ml and HBcAb negative), and 10.4% (*n* = 77) demonstrated sufficient vaccination-derived immunity (HBsAb >10 mIU/ml and HBcAb negative). Natural immunity markers (HBcAb positive) were present in 24.8% (*n* = 183) of HBsAg-negative individuals. Geographic variation was substantial, much higher in South Asian and Northern African migrants compared to those from Sub-Saharan Africa. Conversely, 36.3% (*n* = 70) of Sub-Saharan African participants demonstrated natural immunity versus 13.9% (*n* = 61) of South Asian and 1.0% (*n* = 1) of Northern African migrants.

Table 3 summarizes HBsAg status and susceptibility, further stratified by vaccination-dependent and natural immunity, by sex, age, area of origin, and passage through Libya. Most HBsAg-positive individuals were male and aged ≥25 years, and from South Asia and Sub-Saharan Africa, with no positive cases from Northern Africa or unknown origin. Passage through Libya was reported in most of the HBsAg-positive individuals. Adjusted analyses reflected these patterns, with higher risk of HBsAg positivity observed among males (odds ratio [OR] = 2.02, 95% confidence intervals [CI] 0.24–16.81), those aged ≥25 years (OR = 1.56, 95% CI 0.73–3.34), individuals from Sub-Saharan Africa compared with South Asia (OR = 2.17, 95% CI 0.99–4.73).

**Table 3:** Risk of HBV serology status by sex, age, area of origin, and route [A], risk of HBV susceptibility among vaccination- [B] and natural-immunity [C] groups, assessed among HBsAg-negative individuals as a dichotomous variable (susceptible if HBsAb ≤10 mIU/ml; not susceptible if HBsAb >10 mIU/ml).^a^

HBV serology status [A]	HBsAg negative, *N* (%)	HBsAg positive, *N* (%)	OR (95% CI) crude	OR (95% CI) adjusted
Sex				
Female (*n* = 39)	38 (97.4%)	1 (2.6%)	1.00	1.00
Male (*n* = 700)	671 (95.9%)	29 (4.1%)	1.64 (0.22–12.38)	2.02 (0.24–16.81)
Age				
<25 years (*n* = 349)	336 (96.3%)	13 (3.7%)	1.00	1.00
≥25 years (*n* = 390)	373 (95.6%)	17 (4.4%)	1.18 (0.56–2.46)	1.56 (0.73–3.34)
Macro area of origin				
South Asia (*n* = 439)	423 (96.4%)	16 (3.6%)	1.00	1.00
Sub-Saharan Africa (*n* = 193)	179 (92.7%)	14 (7.3%)	2.07 (0.99–4.33)	2.17 (0.99–4.73)
Northern Africa (*n* = 105)	105 (100.0%)	0 (0.0%)	—	—
Unknown (*n* = 2)	2 (100.0%)	0 (0.0%)	—	—
Libya passage				
No (*n* = 229)	227 (99.1%)	2 (0.9%)	1.00	1.00
Yes (*n* = 510)	482 (94.5%)	28 (5.5%)	6.59 (1.56–27.92)	6.90 (1.60–29.76)

HBcAb negative [B] (vaccination-dependent)	Not susceptible (*n* = 77), *N* (%)	Susceptible (*n* = 449), *N* (%)	OR (95% CI) crude	OR (95% CI) adjusted
Sex				
Female (*n* = 19)	6 (31.6%)	13 (68.4%)	1	1
Male (*n* = 507)	71 (14.0%)	436 (86.0%)	2.83 (1.04–7.70)	4.08 (1.34–12.44)
Age				
<25 years (*n* = 263)	49 (18.6%)	214 (81.4%)	1	1
≥25 years (*n* = 263)	28 (10.6%)	235 (89.4%)	1.92 (1.17–3.17)	2.38 (1.40–4.04)
Macro area of origin				
South Asia (*n* = 344)	46 (13.4%)	298 (86.6%)	1	1
Sub-Saharan Africa (*n* = 77)	9 (11.7%)	68 (88.3%)	1.17 (0.54–2.50)	1.61 (0.71–3.63)
Northern Africa (*n* = 104)	22 (21.2%)	82 (78.8%)	0.58 (0.33–1.01)	0.55 (0.31–0.98)
Unknown (*n* = 1)	0 (0.0%)	1 (100.0%)	—	—
Libya passage				
No (*n* = 169)	23 (13.6%)	146 (86.4%)	1	1
Yes (*n* = 357)	54 (15.1%)	303 (84.9%)	1.52 (0.92–2.50)	1.57 (0.93–2.66)

HBcAb positive [C] (natural immunity)	Not susceptible (*n* = 133), *N* (%)	Susceptible (*n* = 50), *N* (%)	OR (95% CI) crude	OR (95% CI) adjusted
Sex				
Female (*n* = 19)	14 (73.7%)	5 (26.3%)	1	1
Male (*n* = 164)	119 (72.6%)	45 (27.4%)	1.06 (0.36–3.11)	1.35 (0.43–4.26)
Age				
<25 (*n* = 73)	49 (67.1%)	24 (32.9%)	1	1
≥25 (*n* = 110)	84 (76.4%)	26 (23.6%)	0.63 (0.33–1.22)	0.65 (0.32–1.33)
Macro area of origin				
South Asia (*n* = 79)	61 (77.2%)	18 (22.8%)	1	1
Sub-Saharan Africa (*n* = 102)	70 (68.6%)	32 (31.4%)	1.55 (0.79–3.03)	1.38 (0.66–2.87)
Northern Africa (*n* = 1)	1 (100.0%)	0 (0.0%)	—	—
Unknown (*n* = 1)	1 (100.0%)	0 (0.0%)	—	—
Libya passage				
No (*n* = 58)	35 (60.3%)	23 (39.7%)	1	1
Yes (*n* = 125)	98 (78.4%)	27 (21.6%)	0.68 (0.34–1.34)	0.61 (0.30–1.25)

a OR and 95% CI, adjusted for sex, age, area of origin, and entry route.

In the vaccination-dependent group, most susceptible individuals were males (OR = 4.08, 95% CI 1.34–12.44), aged ≥25 years (OR = 2.38, 95% CI 1.40–4.04), and to same extent also for those from Sub-Saharan Africa compared with South Asia (OR = 1.61, 95% CI 0.71–3.63) and those who transit through Libya (OR = 1.57, 95% CI 0.93–2.66). In the natural-immunity group, most susceptible individuals were males (OR = 1.35, 95% CI 0.43–4.26) and from Sub-Saharan Africa (OR = 1.38, 95% CI 0.66–2.87), while lower risk for those aged ≥25 years (OR = 0.65, 95% CI 0.32–1.33) and passage through Libya (OR = 0.61, 95% CI 0.30–1.25). Across all regression models VIF values were below 2.5, indicating no meaningful multicollinearity (maximum VIF 1.88 in the full susceptibility model; maximum VIF 1.40 in the active infection model; mean VIF ≤1.52 across all models).

Analysis of vaccination protocols (Table S3) in countries of origin shows heterogeneity in both the timing of inclusion of HBV vaccination in national vaccination schedules and the provision of a birth dose. The introduction of universal vaccination has spanned for over three decades: a few countries adopted early (e.g. Gambia in 1986, Egypt in 1992), whereas many Sub-Saharan African and South Asian countries implemented programs only after 2000 (e.g. Nigeria in 2004, Bangladesh in 2005), or even more recently (e.g. Somalia and Chad in 2013).

## Discussion

Our study reveals a dual burden of HBV among newly arrived asylum seekers in Italy: an HBsAg prevalence of 4.1%, indicating substantial active infection burden, and high susceptibility rates, with 70.4% having inadequate immunity (HBsAb <10 mIU/ml). We also found high heterogeneity of HBV epidemiology across origin regions and an expected strong influence of transit conditions, particularly passage through Libya. While the direction of the association between Libya transit and HBsAg positivity is consistent with prior literature documenting the elevated infectious disease burden in Libyan detention and transit settings [17, 18], and is biologically plausible, the magnitude of the adjusted estimate (OR 6.90, 95% CI 1.60–29.76) should be interpreted with caution given the limited number of HBsAg-positive individuals who did not transit Libya (*n* = 2).

The observed HBsAg seroprevalence of 4.1% is on the one hand substantially higher than the <1% reported in the Italian general population [3], yet generally lower than rates in migrants’ source countries. Bangladesh and Pakistan, representing the countries of origin of 55.5% of our cohort members, report general population prevalence of approximately 6% and 3%, respectively [19]. Sub-Saharan African countries, particularly West African nations like Côte d‘Ivoire, Nigeria, and Guinea, report rates ranging from 5% to 10%, while North African countries report lower rates, around 3% [1]. This pattern of migrant populations showing HBsAg prevalence intermediate between their countries of origin and the host country confirms previous reports across Europe [3, 6, 18, 20, 21]. It likely reflects factors such as migration-related selection bias (the “healthy migrant effect”), age distribution of migrant populations, and the differences in infection prevalence across source countries. Noteworthy passage through Libya was strongly associated with HBsAg positivity in our study, with migrants reporting this route demonstrating nearly seven-fold increased odds of infection, consistent with the literature [22, 23].

It is noteworthy that the overall HBV prevalence of 4.1% observed in our cohort is lower than rates reported in some other Italian and European screening studies of asylum seekers and migrants, where prevalences of 6%–12% have been described, particularly in cohorts with higher proportions of West and Central African participants [24]. Several factors may explain this difference, but mainly that our cohort is composed predominantly of South Asian migrants (59.4%), characterized by lower HBV prevalence than West African populations [18]. Sub-Saharan African migrants exhibited the highest HBsAg prevalence, exceeding 7%, closely approximating endemic levels in their countries of origin and representing a two-fold increased prevalence compared to South Asian migrants. This high prevalence reflects the hyperendemic nature of HBV in Sub-Saharan Africa, where perinatal and early childhood transmission remain dominant [25, 26]. Notably, more than one-third demonstrated serological evidence of natural immunity (HBcAb positive), which was the highest proportion among all geographic groups, indicating that most infections likely occurred during childhood in endemic settings rather than during migration. South Asian migrants demonstrated a low HBsAg prevalence aligning with the corresponding values of these countries [19, 27] and the rates previously reported in these migrants [18, 28]. A critical clinical consideration for HBsAg-positive individuals from Pakistan is the risk of hepatitis delta virus (HDV) co-infection. Pakistan carries one of the highest global burdens of HDV, with estimated prevalence of around 15% among HBsAg-positive individuals [29]. While HDV testing was not available during this study period, screening protocols for asylum seekers have incorporated systematic anti-HDV antibody testing for all HBsAg-positive individuals from 2025 onwards. No cases of HBsAg positivity were found among Northern African migrants. While both areas have implemented universal HBV vaccination programs, the timing differed substantially: Northern African countries established programs in the early-to-mid 1990s, whereas South Asian countries implemented theirs a decade later. In both regions, more than 60% of migrants were found susceptible to infection, indicating that while active infection rates are low, vaccination coverage gaps persist and catch-up programs would be beneficial. In Italy, the PNPV explicitly provides for free HBV vaccination of susceptible migrants and household contacts of HBsAg-positive individuals, and national screening guidelines recommend systematic HBV testing at the initial health assessment, active recall of individuals to outpatient clinics, and on-site vaccination at reception facilities where logistically feasible. However, well-documented implementation challenges, including fragmented reception center models, regional heterogeneity in policy application, language and cultural barriers, and absence of systematic follow-up to ensure completion of multi-dose vaccination schedules, limit the real-world effectiveness of these provisions. Nonetheless, we adhered to the Local Health Authorities (AUSL) framework, and to overcome language and cultural barriers we employed cultural mediators throughout the process, from the initial screening to the vaccination proposal.

Age emerged as a determinant of HBV susceptibility, particularly among individuals without evidence of prior exposure (HBcAb negative). In this vaccination-dependent group, participants aged 25 years and older were at higher odds of susceptibility (OR = 2.38, 95% CI 1.40–4.04) compared to their younger counterparts. This age-related increase in HBV vulnerability has been observed in other refugee and migrant populations [6, 30, 31].

Given the age distribution of our sample, the largest proportion (51.3%) falls within the 21–29 age range, meaning most participants were born approximately between 1990 and 2002. Participants aged 25 and older at screening (52.8% of the cohort) were predominantly born before vaccination programs were established in most countries of origin. Consequently, this older subgroup had limited opportunity for vaccination during childhood and, in the absence of endemic exposure, remains susceptible to infection. Bangladesh introduced HBV vaccination in 2005, Pakistan in 2002, and most represented Sub-Saharan African countries between 2002 and 2008, some even later, meaning virtually all participants from these areas were born before program implementation. In contrast, Northern African countries introduced vaccination substantially earlier (between 1992 and 2001), partly explaining the absence of HBsAg positivity in this group [32, 33]. Moreover, the relationship between vaccination program implementation and individual protection extends beyond birth year alone. A first critical distinction exists between countries that administered a dose at birth, essential for preventing perinatal HBV transmission, and those that did not [34, 35]. Nominal program existence does not guarantee per se equitable, and therefore effective, coverage, as heterogeneity in vaccine access is also driven by regional disparities between urban and rural areas, socioeconomic factors, and healthcare infrastructure limitations [36, 37]. The absence of birth doses in most programs further compounds this issue, as infants born outside health facilities or in areas with limited healthcare access may miss the entire vaccination series [38].

The male predominance of susceptibility in our study may reflect several factors related to the migration process itself. Our cohort was overwhelmingly male, likely representing a selected subpopulation, whose specific risks and conditions experienced before and during migration may differ by sex in ways that influence both exposure and health-seeking behavior [39, 40].

### Strengths and limitations

This study has several strengths. Firstly, the large, consecutive cohort reflects real-world screening practices, and the quantitative three-marker serological panel (HBsAg, HBcAb, and HBsAb titers) enabled differentiation between active infection, natural and vaccine-induced immunity, and threshold-based assessment of protection adequacy. Additionally, data were collected using a standardized regional tool (Migrant Checklist), which ensured consistency throughout the multi-year study period. Lastly, detailed self-reported migration history allowed us to accurately map each migrant’s journey and stratify their risk. While direct generalization to other Italian or European settings requires caution given the single-province design, the epidemiological patterns observed are consistent with broader trends documented in asylum-seeker populations across Europe.

There are some limitations to consider. The single-province setting, restricts generalizability, as well as the predominantly male cohort. Self-reported migration data may be affected by recall bias, and the cross-sectional design captures serological status at one timepoint, precluding assessment of incident infections or temporal changes in immunity. Absence of documented vaccination history, viral load data for HBsAg-positive cases, and HDV co-infection screening reduces clinical characterization. Vaccination completion data were not systematically available, as follow-up tracking after the initial health assessment was not standardized during the study period, precluding assessment of program effectiveness. Grey literature on source-country vaccination policies was likely incomplete, given the difficulty in finding official open-access documents, limiting attribution of susceptibility patterns.

## Conclusions

These findings confirm a substantial burden of HBV susceptibility and undiagnosed infection among asylum seekers in northern Italy. This represents a dual public health responsibility: undetected infections and unvaccinated susceptible individuals face risks of long-term complications, while serving as potential vectors for ongoing transmission within host communities. The implementation of systematic screening and catch-up vaccination programs for newly arrived migrants is therefore a public health imperative. Such programs require adequate resources, cultural mediation services, and integration into existing healthcare pathways to ensure accessibility and continuity of care.

Future research should focus on longitudinal studies tracking post-vaccination antibody responses and seroconversion, and prospective investigations of HBV exposure during migration, especially through Libya, including detention conditions, healthcare access, and behavioral risks. Further research is needed on barriers to childhood vaccination in high-burden countries, cost-effectiveness of universal versus risk-based screening and accelerated vaccination schedules, and booster strategies to address the substantial proportion of migrants with waning natural or vaccine-induced immunity.

## Supplementary Material

ckag188_Supplementary_Data

## Data Availability

The data presented in this study are available on reasonable request from the corresponding author. The data are not publicly available due to privacy and legal restrictions. Key points In migrant populations, HBV epidemiology showed strong heterogeneity by region of origin, with Sub-Saharan Africa exhibiting the highest prevalence, and transit through Libya being strongly associated with increased infection risk. The implementation of quantitative three-marker serological panel (HBsAg, HBcAb, and HBsAb titers) enables accurate differentiation between active infection, natural and vaccine-induced immunity, and threshold-based assessment of protection adequacy. This study highlights the public health imperative for systematic HBV screening and catch-up vaccination programs for newly arrived migrants to reduce ongoing transmission and prevent long-term complications.
