# Supplementary material for: Hepatitis B screening among asylum seekers: a retrospective cohort study in Modena, Northern Italy
Source: Eur J Public Health. 2026 Oct 21;36(6):ckag188. doi: 10.1093/eurpub/ckag188 (PMC13686764; doi:10.1093/eurpub/ckag188)
Supplement: Supplementary file 1 [file ckag188_supplementary_data.zip › Files_BKup/15-Oct-2026_043409_ejph-2026-02-om-0134-File003.docx]

**Supplementary Table S1.** Categorization of countries by area of origin.

| **Area of origin** | **Countries** |
| --- | --- |
| Northern Africa | Algeria, Egypt, Morocco, Tunisia |
| Sub-Saharan Africa | Burkina Faso, Cameroon, Chad, Congo, Ivory Coast, Gambia, Ghana, Guinea, Mali,  Nigeria, Senegal, Sierra Leone, Somalia, Sudan |
| Asia | Afghanistan, Azerbaijan, Bangladesh, India, Iraq, Pakistan, Syria, Turkey |

**Supplementary Table S2.** Number (n) and percentage (%) of subjects divided by country of origin.

| **Country of origin** | **n** | **%** |
| --- | --- | --- |
| Bangladesh | 303 | 41.00 |
| Pakistan | 107 | 14.48 |
| Egypt | 73 | 9.88 |
| Côte d’Ivoire | 53 | 7.17 |
| Tunisia | 26 | 3.52 |
| Gambia | 22 | 2.98 |
| Nigeria | 20 | 2.71 |
| Guinea | 19 | 2.57 |
| Cameroon | 17 | 2.30 |
| Ghana | 16 | 2.17 |
| Somalia | 15 | 2.03 |
| Mali | 14 | 1.89 |
| Turkey | 11 | 1.49 |
| Afghanistan | 8 | 1.08 |
| Burkina Faso | 8 | 1.08 |
| Iraq | 5 | 0.68 |
| Morocco | 5 | 0.68 |
| Senegal | 5 | 0.68 |
| Azerbaijan | 2 | 0.27 |
| Unknown | 2 | 0.27 |
| Syria | 2 | 0.27 |
| Algeria | 1 | 0.14 |
| Chad | 1 | 0.14 |
| Congo | 1 | 0.14 |
| India | 1 | 0.14 |
| Sierra Leone | 1 | 0.14 |
| Sudan | 1 | 0.14 |

**Supplementary Table S3.** Vaccination protocols in different countries of origin.

| **Country** | **Vaccination mandatory** | **Intro Year (EPI)** | **Birth Dose** | **Notes** | **Reference/link** |
| --- | --- | --- | --- | --- | --- |
| Afghanistan | No (recommended for infants) | 2006 | No (not universal) | Afghanistan introduced HBV vaccine into its routine infant immunization program in 2006. As of 2014, a birth dose policy was limited to health facility deliveries (not nationwide). | PMC3955263 |
| Algeria | Yes (infant immunization in schedule) | 2001 | Yes (at birth) | Algeria’s national schedule includes HBV for newborns (1st dose at birth). (Exact introduction year not explicitly documented; HBV was added in the early 2000s) | 10.15585/mmwr.mm7229a2  https://data.unicef.org/wp-content/uploads/cp/immunisation/dza.pdf |
| Armenia | Yes (infants, universal) | 1999 | Yes (at birth) | Armenia adopted universal infant HBV vaccination in 1999. The HBV birth dose has been part of the program (the monovalent birth dose was maintained even after switching to pentavalent vaccine). | <https://armenpress.am/en/article/1089152>  https://immunizationeconomics.org/wp-content/uploads/2017/12/BRIEF19.pdf#:~:text=The%20government%20introduced%20the%20hepatitis,through%20UNICEF%29%2C%20with |
| Azerbaijan | Yes (infants, universal) | 2001 | Yes (at birth) | Azerbaijan introduced universal HBV vaccination for newborns in 2001, with the first dose given at birth (in 2012, ≥96% coverage). | 10.1111/1469-0691.12535 |
| Bangladesh | Yes (infants, routine) | 2005 | No | Bangladesh introduced HBV vaccine into the infant EPI in 2005. A birth dose is not included in the schedule (infants receive first HBV dose at 6 weeks). | http://www.who.int/immunization/monitoring_surveillance/data/en/ https://www.idhsdata.org/idhs/vaccines.shtml |
| Benin | Yes (infants, routine) | 2002 | No | Benin added HBV to its routine infant schedule in 2002. No birth dose is given in the national schedule (first dose in infancy). | http://www.who.int/immunization/monitoring_surveillance/data/en/ https://www.idhsdata.org/idhs/vaccines.shtml |
| Burkina Faso | Yes (infants, routine) | 2006 | No | Burkina Faso introduced HBV vaccine in 2006 as part of routine infant immunizations. It does not administer an HBV birth dose (first dose given later in infancy). | http://www.who.int/immunization/monitoring_surveillance/data/en/ https://www.idhsdata.org/idhs/vaccines.shtml |
| Cameroon | Yes (infants, routine) | 2005 | No | Cameroon’s EPI began including HBV vaccine in 2005. There is no birth dose in the schedule (doses start at 6 weeks of age). | http://www.who.int/immunization/monitoring_surveillance/data/en/ https://www.idhsdata.org/idhs/vaccines.shtml |
| Chad | Yes (infants, routine) | 2013 | No | Chad introduced HBV (via pentavalent vaccine) by 2013, achieving universal rollout by 2014. The schedule does not include a birth dose (first dose given in infancy). (Chad was among the last Gavi-supported countries to introduce HBV in EPI, completed by 2014.) | http://www.who.int/immunization/monitoring_surveillance/data/en/ https://www.idhsdata.org/idhs/vaccines.shtml |
| Congo (DRC) | Yes (infants, routine) | 2007 | No | Democratic Republic of Congo introduced HBV (pentavalent) in 2007 as part of routine infant immunization. No HBV birth dose is given in the DRC’s schedule. | http://www.who.int/immunization/monitoring_surveillance/data/en/ https://www.idhsdata.org/idhs/vaccines.shtml |
| Côte d’Ivoire | Yes (infants, routine) | 2003 | No | Côte d’Ivoire added HBV vaccine to the infant immunization program in 2003. A birth dose is not yet part of the national schedule (doses start at 6, 10, 14 weeks). | http://www.who.int/immunization/monitoring_surveillance/data/en/ 10.1016/j.vaccine.2008.03.018 |
| Egypt | Yes (infant vaccinations compulsory) | 1992 | Yes (phased introduction from ~2015) | Egypt incorporated HBV into its compulsory childhood vaccination list in 1992. A birth dose has been introduced in a phased manner (initially in select governorates starting 2015), aiming for nationwide newborn coverage. | 10.1186/s43066-024-00362-3 |
| Gambia | Yes (infants, universal) | 1986 (phased to 1990) | Yes (since 1990s) | The Gambia was the first African nation to implement HBV vaccination – begun in 1986 as a phased national trial, achieving nationwide infant coverage by 1990. An HBV birth dose has been part of the program since the 1990s; Gambia introduced a birth dose early and has maintained high HepB birth dose coverage. | 2822233 30931434 |
| Ghana | Yes (infants, routine) | 2002 | No | Ghana introduced HBV vaccine in 2002 as part of the routine infant EPI (via pentavalent vaccine). No birth dose is given in Ghana’s schedule (HBV doses begin at 6 weeks). | http://www.who.int/immunization/monitoring_surveillance/data/en/ 10.1016/j.vaccine.2008.03.018 |
| Guinea | Yes (infants, routine) | 2006 | No | Guinea incorporated HBV vaccine into its infant schedule in 2006. A birth dose is not included (HBV vaccination starts in early infancy). | http://www.who.int/immunization/monitoring_surveillance/data/en/ 10.1016/j.vaccine.2008.03.018 |
| India | No (voluntary, part of routine program) | 2002 (phased; nationwide by 2011) | Yes (partial, since 2008) | India began HBV vaccination in 2002 in pilot states and expanded nationwide by 2011. A birth dose is recommended – introduced in select states around 2008 – though coverage varies (the first dose for many infants is given at birth in hospital settings). | http://www.who.int/immunization/monitoring_surveillance/data/en/ 10.1016/j.vaccine.2008.03.018 |
| Iraq | Yes (infant vaccinations compulsory) | 1994 | Yes (at birth) | Iraq adopted WHO’s recommendation and added HBV vaccine to its EPI in 1993–1994, making it part of the infant schedule. Newborns receive the first dose at birth as part of the national policy (universal birth dose). | 10.4103/IRJCM.IRJCM_5_20 |
| Liberia | Yes (infants, routine) | 2008 | No | Liberia introduced the pentavalent vaccine (including HBV) in 2008 for all infants. The immunization schedule does not include a hepatitis B birth dose (doses are given starting at 6 weeks). | https://extranet.who.int/countryplanningcycles/sites/default/files/country_docs/Liberia/liberia_cmyprev_25_03_2014.pd |
| Mali | Yes (infants, routine) | 2002 | No | Mali added HBV vaccine to its routine infant immunizations in 2002. No birth dose is given (HBV vaccination begins at 6, 10, 14 weeks). | http://www.who.int/immunization/monitoring_surveillance/data/en/ 10.1016/j.vaccine.2008.03.018 |
| Morocco | Yes (infant vaccinations compulsory) | 1999 | Yes (at birth) | Morocco introduced HBV vaccine in 1999 as part of EPI. From the outset, Morocco included a hepatitis B birth dose – the first dose is given at birth (with HBV coverage >90% in infants). | http://www.who.int/immunization/monitoring_surveillance/data/en/ 10.1016/j.vaccine.2008.03.018 |
| Mauritania | Yes (infants, routine) | 2004 | No | Mauritania’s EPI added HBV vaccine around 2004 with Gavi support (proposal submitted 2003). The schedule currently has no HBV birth dose (infants receive HBV starting at 6 weeks). | http://www.who.int/immunization/monitoring_surveillance/data/en/ 10.1016/j.vaccine.2008.03.018 |
| Nigeria | Yes (infants, routine) | 2004 | Yes (at birth) | Nigeria introduced HBV vaccine in 2004 as part of routine infant immunization. The national schedule includes a birth dose at delivery (HBV0), although actual coverage of the timely birth dose is limited. | http://www.who.int/immunization/monitoring_surveillance/data/en/ 10.1016/j.vaccine.2008.03.018 |
| Pakistan | Yes (infants, routine) | 2002 | No (pilot only) | Pakistan introduced HBV vaccine in 2002 in its infant EPI (as part of pentavalent). A birth dose is not yet universally implemented (as of 2014, only pilot programs in some provinces). Infants generally receive HBV starting at 6 weeks. | http://www.who.int/immunization/monitoring_surveillance/data/en/ 10.1016/j.vaccine.2008.03.018 |
| Senegal | Yes (infants, routine) | 2004 | Yes (since 2016) | Senegal added HBV to the infant schedule in 2004. In 2016, Senegal became one of the first in West Africa to introduce a hepatitis B birth dose into the schedule, administering the first dose within 24 hours of birth. | http://www.who.int/immunization/monitoring_surveillance/data/en/ 10.1016/j.vaccine.2008.03.018 |
| Sierra Leone | Yes (infants, routine) | 2007 | No | Sierra Leone introduced the pentavalent vaccine (including HBV) in 2007 after the civil war. The current schedule does not include a birth dose of HBV (doses are given at 6, 10, 14 weeks). | https://www.gavi.org/sites/default/files/document/2021/cMYP%20Sierra%20Leone%202017-2021.pdf |
| Syria | Yes (infant vaccinations compulsory) | 1993 | Yes (at birth) | Syria was among the first Middle Eastern countries to introduce HBV vaccine, adding it to the infant program in 1993. A birth dose for newborns is included in the schedule, although coverage is challenged by home births (no official estimate for timely birth dose due to ~22% home deliveries). | https://www.nitag-resource.org/sites/default/files/8bfb799f04a866af145e3f5cdbc786f57ad0785b_1.pdf |
| Somalia | Yes (infants, routine) | 2013 | No | Somalia introduced the pentavalent vaccine in 2013 as part of its routine infant immunization program. There is no hepatitis B birth dose in the immunization schedule (given low institutional delivery rates). | https://www.emro.who.int/somalia/news/vaccines-are-saving-millions-of-lives-of-children-in-somalia.html |
| Sudan | Yes (infants, routine) | 2004 | No | Sudan (Khartoum government) incorporated HBV vaccine in 2004 into the infant EPI (as part of pentavalent). A birth dose is not administered (HBV doses start at 6 weeks). (South Sudan introduced pentavalent later, in 2014.) | http://www.who.int/immunization/monitoring_surveillance/data/en/ 10.1016/j.vaccine.2008.03.018 |
| Togo | Yes (infants, routine) | 2008 | No (planned) | Togo introduced HBV (with Hib) in 2008 as part of the EPI. A hepatitis B birth dose has been under consideration – as of the 2016–2022 plan, Togo aimed to add a birth dose, but it is not yet fully implemented in the routine schedule. | https://www.acceleratehss.org/wp-content/uploads/2023/11/Accelerator_Togo-Report-2023_web-version.pdf |
| Tunisia | Yes (infant vaccinations compulsory) | 1995 | Yes (since 2006) | Tunisia incorporated HBV into its national immunization program in 1995. Initially the series was given at 2, 3, 9 months; a birth dose for newborns was later introduced in 2006. Tunisia mandates childhood vaccines, including hepatitis B. | 10.1093/eurpub/ckz187.156 |
| Turkey | Yes (infant vaccinations compulsory) | 1998 | Yes (at birth) | Turkey launched universal infant hepatitis B immunization in 1998. All newborns receive the first HBV dose within 24 hours of birth, followed by two additional doses in infancy. Hepatitis B vaccination has since been routine for all Turkish infants. | https://www.worldgastroenterology.org/publications/e-wgn/e-wgn-expert-point-of-view-articles-collection/viral-hepatitis-elimination-program-in-trkiye |
